# A registration trend in eyelid skin cancers and associated risk factors in Iran, 2005–2016

**DOI:** 10.1186/s12885-023-11414-z

**Published:** 2023-09-30

**Authors:** Sare Safi, Mohadese Ahmadzade, Saeed Karimi, Mohammad Esmaeil Akbari, Hamidreza Rouientan, Mohammad Abolhosseini, Mozhgan Rezaei Kanavi, Zahra Khorrami

**Affiliations:** 1 Ophthalmic Epidemiology Research Center, Research Institute for Ophthalmology and Vision Science, Shahid Beheshti University of Medical Sciences, Tehran, Iran; 2 Department of Optometry, School of Rehabilitation, Shahid Beheshti University of Medical Sciences, Tehran, Iran; 3 Ophthalmic Research Center, Research Institute for Ophthalmology and Vision Science, Shahid Beheshti University of Medical Sciences, Tehran, Iran; 4 Cancer Research Center, Shahid Beheshti University of Medical Sciences, Tehran, Iran; 5 Ocular Tissue Engineering Research Center, Research Institute for Ophthalmology and Vision Science, Shahid Beheshti University of Medical Sciences, Tehran, Iran

**Keywords:** Eyelid skin cancers, Age-standardized incidence rates, Risk factors, Iran

## Abstract

**Background:**

Eyelid skin cancers are the most prevalent ophthalmic malignancies. This study aimed to evaluate the association of the Human Development Index (HDI) and lifestyle risk factors with eyelid skin cancers in Iran.

**Methods:**

This ecological study analyzed the data collected from the Iranian National Population-based Cancer Registry (2005–2016). The data on provincial-level eyelid skin cancer risk factors were obtained from national sources. The association between provincial HDI and lifestyle risk factors with the prevalence of eyelid skin cancers was assessed.

**Results:**

The mean 12-year age-standardized incidence rate (ASIR) of eyelid skin cancers was 16.22 per 100,000 (9,104 cases). The overall ASIR showed an upward trend with an estimated annual average increase of 0.006 per year. There were positive correlations between the prevalence of overall eyelid skin cancers and provincial HDI, smoking, and obesity (*r* = 0.32, 0.42, and 0.37, respectively). In multivariate analysis, obesity/overweight remained a positive predictor for high prevalence of total eyelid skin cancers (OR = 1.97, 95%CI = 1.08–3.58, *P* = 0.026), carcinoma (2.10, 1.15–3.83, *P* = 0.015), and basal cell carcinoma (1.48, 0.99–2.20, *P* = 0.054).

**Conclusions:**

An increasing trend in ASIR of eyelid skin cancers was observed in more than a decade in Iran which was positively associated with provincial HDI and prevalence of obesity. The findings of the study highlight the importance of promotional programs for preventing obesity/overweight and appropriate allocation of screening facilities based on the HDI level.

## Background

Eyelid skin cancers are the most common malignant neoplasms in ophthalmic practice [1, 2]. Despite having a very low rate of mortality [3] these cancers may lead to significant changes in facial appearance and consequent psychological distress. Furthermore, their local spread to the orbital area or distant metastasis may pose additional challenges to the treatment process [4, 5].

Globally the incidence of malignant eyelid lesions is increasing with the variable distribution [6–8]. The incidence of age-adjusted eyelid malignancies ranges from 5.1 in Singapore to 6.5 in Taiwan and 15.7 per 100,000 population per year in Minnesota, United States [7, 9, 10]. The 9-year prevalence of eyelid skin cancers was 145.1 per 100,000 population in the United States (2010–2018). Cancer-specific prevalence ranged from 87.9 in basal cell carcinomas (BCC) to 11.1 in squamous cell carcinomas (SCC) and 4.1 per 100,000 population in malignant melanomas [11]. The age-standardized incidence rates (ASIR) of eyelid skin cancers increased from 0.27 in 1999 to 0.61 per 100,000 population in 2016, with an annual percent change of 4.94% in South Korea [12].

The challenge of cancers in the world constantly changes as the countries experience crucial alterations, appraised by Human Development Index (HDI) [13]. It was estimated that the incidence rate of cancers in more developed countries is 2.5 folds of that in low developed countries in 2020 [14]. Iran as one of the most populated countries in the Eastern Mediterranean Region (EMR) [15] ranked the second in fast aging countries in the world [16] and confronting demographic and epidemiologic transition. This transition involved a change of risk factors contributed to cancers like eyelid skin cancers, with a considerable burden on health systems [17]. A number of factors have been suggested as possible risk factors for the incidence of eyelid skin cancers, including aging, male gender, smoking and ultraviolet (UV) exposure [11]. However, studies on the effects of a combination of lifestyle, socioeconomic and environmental risk factors on the incidence of eyelid skin cancers based on HDI level are limited. Hence, this study was performed to investigate the 12-year spatial trend of the most common ocular cancers, eyelid skin cancers, and the associated risk factors in Iran.

## Methods

### Study design and data collection

In this ecological study, the eyelid skin cancer data between 2005 and 2016 was extracted from the Iranian National Population-based Cancer Registry (INPCR). This registry was initiated in 1986 in Iran with the collaboration of the Iranian Ministry of Health and Medical Education and local universities [18]. The carcinomas (International classification of Diseases codes 801–805, 807, 808, 809, 811, 812, 839, 843), adenocarcinoma (814, 820–824, 826, 840–841, 848, 856), melanoma (872–874 and 876–878), and other eyelid skin cancers (880–898, 901, 907, 914, 931, 959, 965, 967–973, 996) were included [19]. The data of 31 provinces were analyzed to evaluate the association of the HDI and lifestyle risk factors with the prevalence of eyelid skin cancers.

### Outcomes and variables

The ASIR and the 3-year prevalence trend of eyelid skin cancers were analyzed at the provincial level. The trends of ASIR were stratified by age groups** (**age < 50 and age ≥ 50 years**),** gender, and cancer morphology (SCC, BCC, adenocarcinoma, melanoma, and others). The HDI data at the provincial level were divided into three categories according to the percentile 33.3 and 66.7 (low: HDI < 0.68, medium: 0.7 > HDI ≥ 0.68, and high: HDI ≥ 0.7) [20].

The lifestyle risk factors were obtained from the STEPS Non-Communicable Disease Risk Factors Survey in 2016 [21]. These risk factors included obesity/overweight [body mass index (BMI) ≥ 25 kg/m^2^], daily cigarette smoking (daily cigarette smoking or past history of smoking), alcohol consumption (drinking alcohol in the past 12 months), hypertension (systolic blood pressure ≥ 140 mmHg or diastolic blood pressure ≥ 90 mmHg), low physical activity (**≤ **600 metabolic equivalent of task according to Global Physical Activity Questionnaire), diabetes (fasting blood glucose ≥ 126 mg/dl, high total cholesterol (total cholesterol > 200 mg/dl), low fruit intake (the consumption of < 2 servings) and low vegetable intake (the consumption of < 3 servings). The levels of UV radiation exposure was extracted from the Iran Meteorological Organization [22].

### Statistical analyses

The incidence rate was standardized based on the Standard World Population (2004) and using a direct method for age groups, gender, and cancer morphology [23]. The incidence trend changes were presented as the estimated annual percentage change (EAPC) of ASIRs and 95% confidence interval (CI). The EAPC is a summative and widely used measure of the ASIR trend over a specified interval. These statistics were calculated by fitting a simple regression model to the logarithm of the ASIRs. The EAPC was estimated using the following equation:

$$\begin{array}{c}\mathrm{y}=\mathrm{a}+\mathrm{\beta x}+\upvarepsilon ,\\ \mathrm{EAPC}=100\times (\mathrm{exp}\left(\upbeta \right)-1)\end{array}$$

The trend in ASIR is reflected in EAPC. If EAPC and lower boundary of the 95% CI are positive, the trend of ASIR will be upward [24].

The 3-year prevalence was assessed as the proportion of persons who had eyelid skin cancers diagnosed within the past 3 years per 100,000 persons in each province. The data was normalized using the Kolmogorov–Smirnov test. Considering the correlation of lifestyle and environmental risk factors with high HDI levels, we further analyzed the association between the 3-year prevalence of eyelid skin cancers and the provincial HDI levels using Spearman's correlation. This correlation was presented by the correlation coefficient (r). Provinces were divided into two groups (high and low prevalence provinces) according to the medians of the 3-year prevalence of total eyelid skin cancers and the most common morphology (SCC and BCC).

Thereafter, the association of lifestyle risk factors with the prevalence of eyelid skin cancers at the provincial level was evaluated using logistic regression analyses to present odds ratios (ORs). The logistic regression models and receiver operating characteristic (ROC) curve were used to predict the risk of eyelid skin cancers. The prediction probabilities of investigated lifestyle risk factors were presented as the area under the curves (AUCs). Statistical analyses were done using the Stata (version 17.0) and SPSS (version 26) statistics software, statistical differences were considered significant at P ≤ 0.05.

## Results

**Fig. 1:**
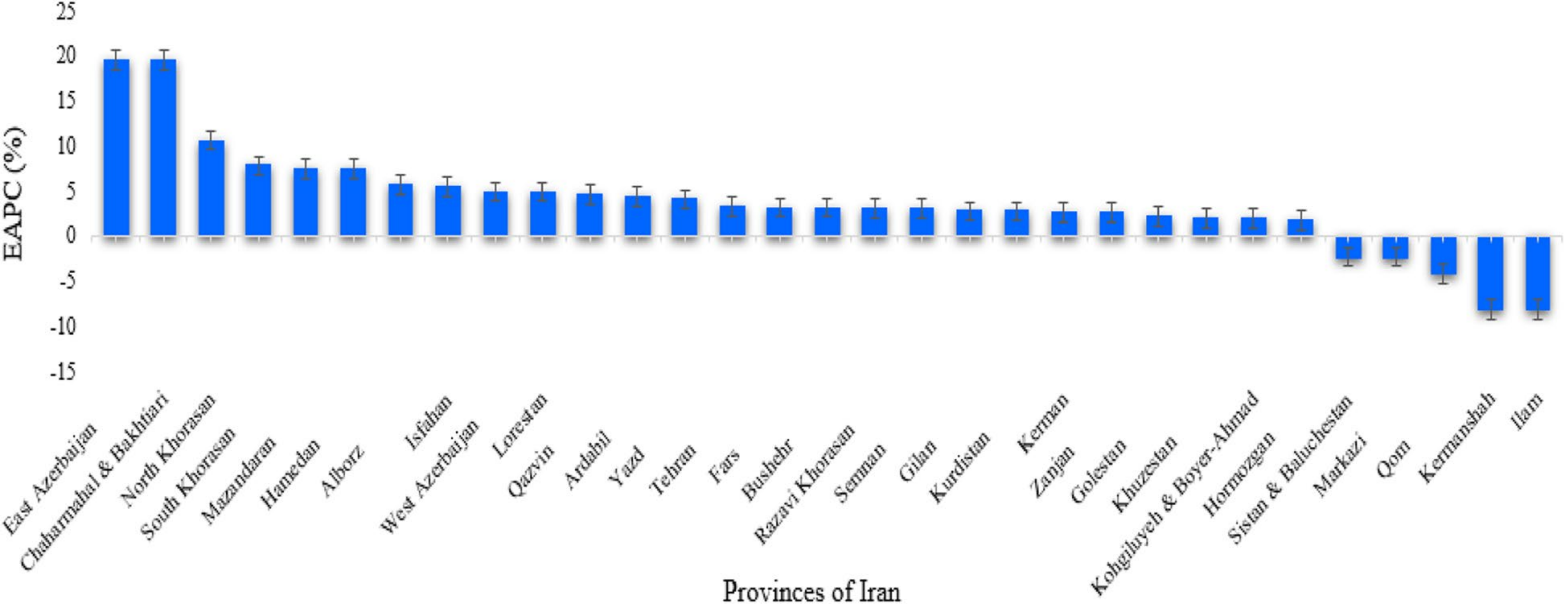
The trends in the EAPC of eyelid skin cancers ASIR by Iran provinces from 2005 to 2016. The trend is upward when the EAPC and the 95% CI are positive; and is downward when EAPC and the 95% CI are negative. EAPC, estimated annual percentage change; ASIR: age-standardized incidence rate

A total of 9,104 eyelid skin cancer cases were identified between 2005 and 2016 in Iran (male = 5,156 and female = 3,948) with an increase of 78.48% from 2005 to 2016. The most common morphological type of eyelid skin tumors in our study was carcinoma (94%), followed by adenocarcinoma (2.75%), and melanoma (1.3%). Almost 88% of carcinomas were BCC, followed by SCC (9.5%) and sebaceous carcinoma (2%). The overall ASIR of eyelid skin cancers was 16.22 per 100,000, which showed an upward trend during the 11-year period by an estimated annual average of 0.006 per year (EAPC = 1.511; 95% CI: 0.00—2.942) (Fig. 1). This increasing trend was detected by all age groups, genders, and morphology types except adenocarcinoma (-26.68%). The highest alteration was observed in the melanoma (733.33%) group and the age less than 50 years category (102.98%) (Table 1). The incident trends in total eyelid skin cancers increased in most areas except five provinces (Ilam, Kermanshah, Qom, Markazi, and Sistan & Baluchestan). East Azerbaijan and Chaharmahal & Bakhtiari had the most EAPC from 2005 to 2016 (Fig. 1).

**Table 1:** The number and age-standardized rate of eye skin cancers by age groups, genders, morphology types, and human development index levels at provincial level in Iran in 2005 and 2016

Characteristics	2005			2016			2005–2016	
	N	Crude rate, per 100,000 pop	ASIR per 100,000 pop	N	Crude rate, per 100,000 pop	ASIR per 100,000 pop	Change in number (%)	EAPC in ASIR (95%CI)
**Overall**	474	0.665	27.23	846	1.058	36.56	78.48	1.511(0.00–2.942)
**Age(year)**								
< 50 year	67	0.109	3.14	136	0.208	4.17	102.98	0.200(-2.955–3.355)
≥ 50 year	407	4.168	24.09	710	5.005	32.39	74.45	1.715(0.200–3.149)
**Gender**								
Male	258	0.712	1.162	485	1.197	1.504	87.9	2.020(-3.825–8.329)
Female	216	0.617	1.091	361	0.916	1.297	67.13	1.410(-4.496–7.573)
**Morphology**								
Carcinomas	451	0.633	26.078	727	0.909	32.347	61.19	0.904(-0.698 – 2.429)
BCC	541	0.767	17.32	700	0.876	27.18	29.39	3.355(1.005–5.759)
Adenocarcinomas	15	0.021	0.831	11	0.014	0.262	-26.68	-0.399(-5.257–4.812)
Melanomas	3	0.004	0.077	25	0.031	1.078	733.33	10.517(4.185–17.351)
Others skin tumors	5	0.007	0.249	83	0.104	2.871	1560	7.681(-22.818–50.080)
**HDI levels**								
Low	83	0.683	10.045	100	0.729	9.399	20.48	0.501(-1.193 -2.224)
Medium	146	0.641	8.723	284	1.119	13.793	94.52	2.737(-0.598–6.290)
High	245	0.675	8.466	462	1.131	13.365	88.57	3.977(0.401–7.681)

*N* Number, *ASR* Age-standardized rate, *EAPC* The estimated annual percentage change, *CI* Confidence interval, *HDI* Human development index, *BCC* Basal cell carcinoma. Percentage change in absolute number was calculated based on the row data. HDI: low, HDI < 0.68; medium, 0.68 ≤ HDI < 0.70; high, HDI ≥ 0.70

### HDI and eyelid skin cancers in 2016

**Fig. 2:**
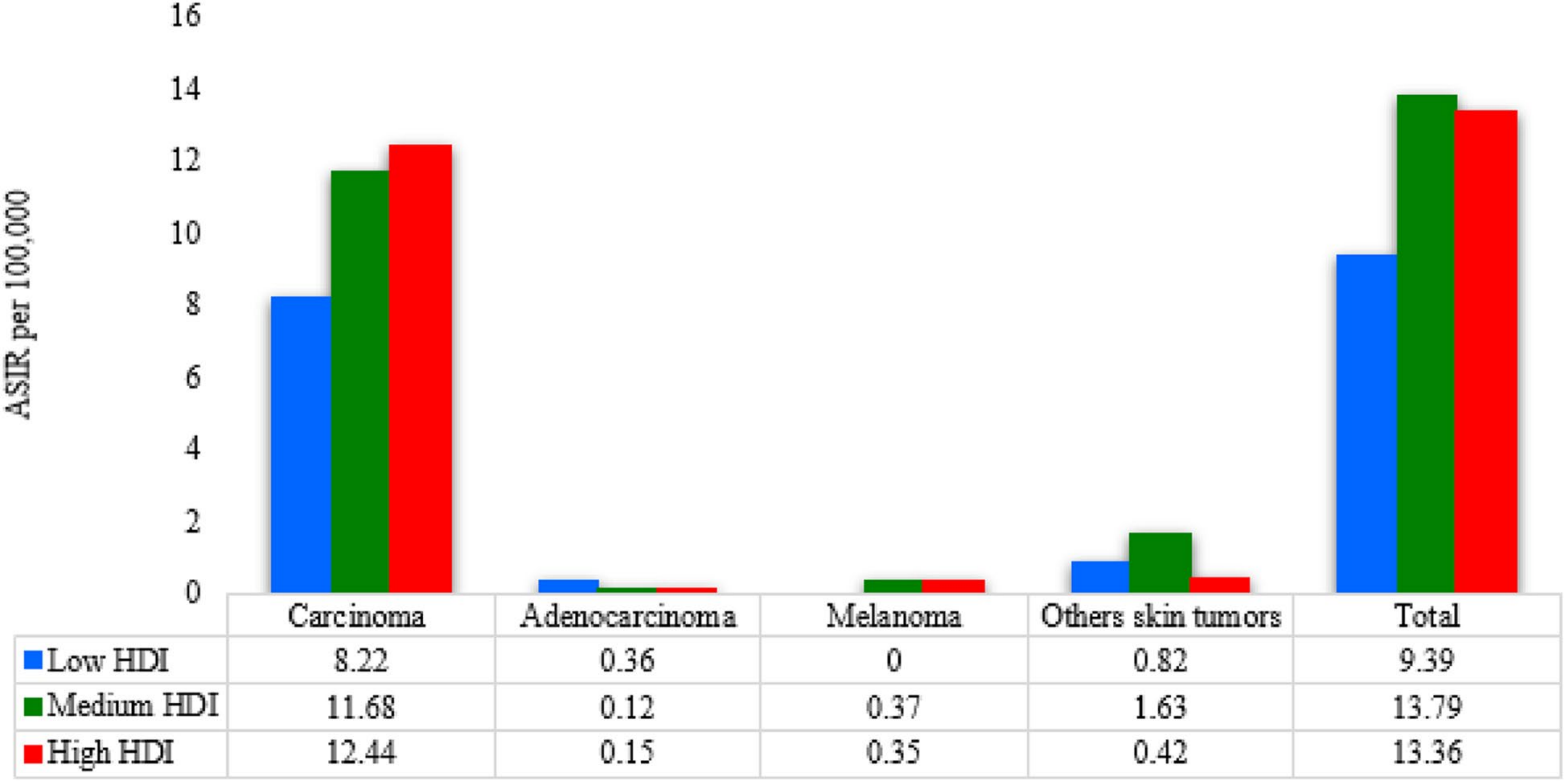
The ASIR of morphology types of eyelid skin cancers stratified by the HDI level in Iran in 2016. The ASIRs of total eyelid skin cancers and carcinoma morphology subtypes are increased in high level of HDI. ASIR: age-standardized incidence rate; HDI: human development index

In 2016, the mean provincial ASIR of total eyelid skin cancers was 1.17 per 100,000 (CV% = 37.49). It ranged from 0.28 per 100,000 in Sistan & Baluchestan to 2.37 per 100,000 in Khorasan Razavi. An increasing trend was observed in all HDI levels. However, this increasing trend only statistically significant in high HDI level, (EAPC = 3.977; 95% CI: 0.401 to 7.681, *P* < 0.05) (Table 1). The ASIR changes of total eyelid skin cancers from 2005 to 2016 in low, medium and high HDI levels were 121.46, 123.02, and 149.12 per 100,000, respectively. The ASIR of eyelid carcinoma was consistently higher in provinces with high HDI levels (Fig. 2).

**Fig. 3:**
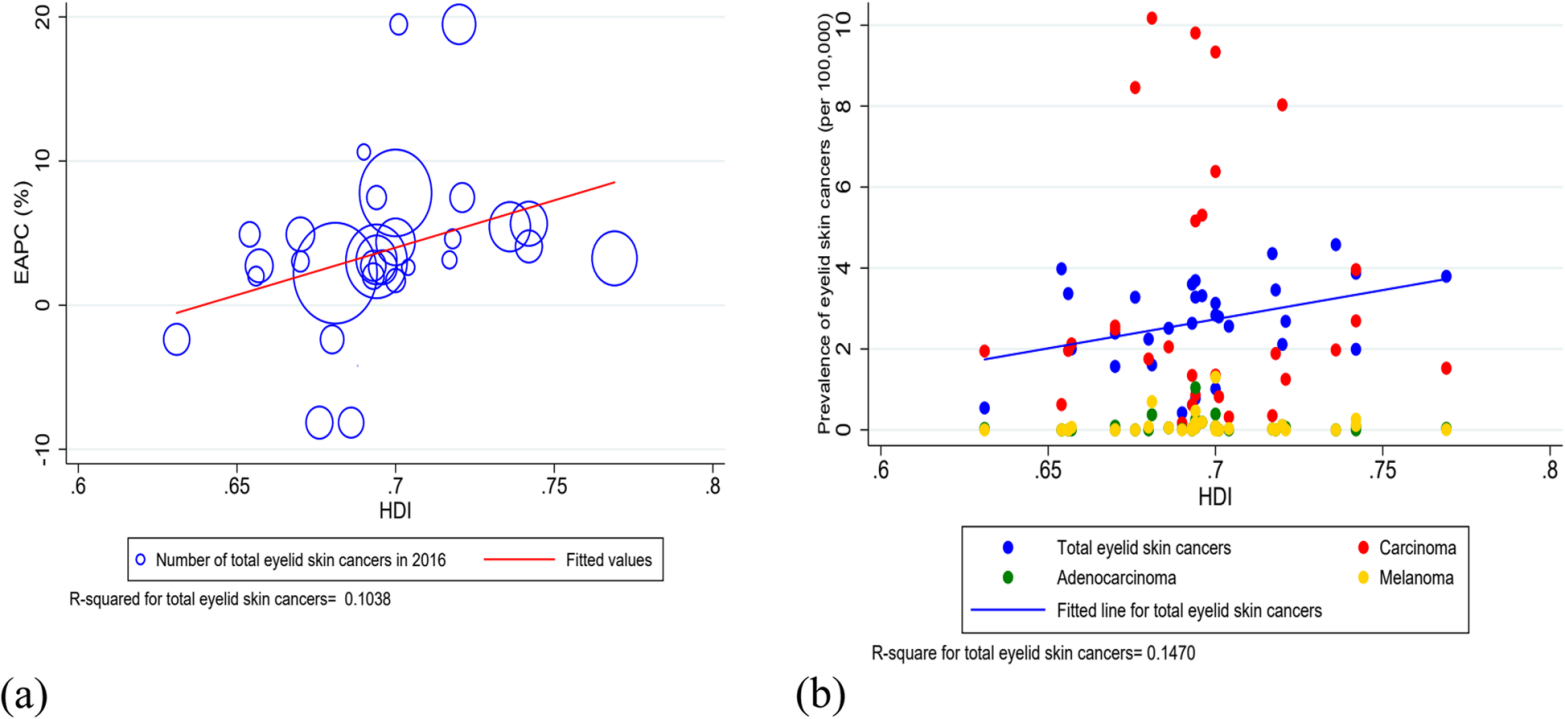
The correlation between HDI in 2016 at the provincial level in Iran and EAPC and prevalence of eyelid skin cancers. There is a positive correlation between HDI and EAPC (**a**), and prevalence (**b**) of eyelid skin cancers. EAPC: estimated annual percentage change; HDI: human development index

The prevalence of total eyelid skin cancers and EAPC were positively correlated with the provincial HDI (*r* = 0.32, *P* = 0.077_for EAPC_, *r* = 0.317, *P* = 0.088_for prevalence of total eyelids skin cancers_) (Fig. 3).

The prevalence of different eyelid skin cancer morphologies was positively correlated with provincial HDI (r _for carcinoma_ = 0.27, r _for melanoma_ = 0.20, r _for adenocarcinoma_ = 0.197), with the positive significant correlation seen in BCC (*r* = 0.37, *p* = 0.034).

### Risk factors and eyelid skin cancers in 2016

**Fig. 4:**
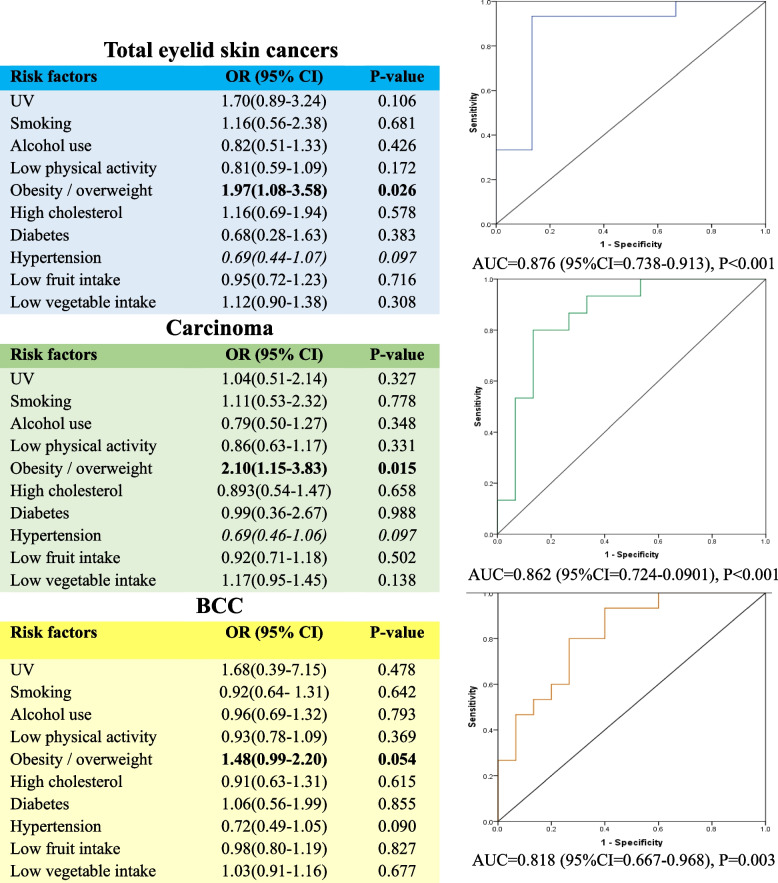
Determination of risk factors for total eyelid skin cancers, carcinoma, and BCC at the provincial level in Iran. A higher prevalence of obesity/overweight is associated with a higher prevalence of eyelid skin total cancers, carcinomas, and BCC. BCC: basal cell carcinoma; ORs: odds ratios; CIs: confidence intervals; AUCs: area under the curves

Table 2 shows the correlation of lifestyle risk factors with the 3-year prevalence of provincial eyelid skin cancers. The total prevalence of eyelid skin cancers was positively correlated with prevalence of smoking (*r* = 0.42, *P* = 0.019) and obesity/overweight (*r* = 0.37, *P* = 0.045). There were positive correlations between carcinomas and smoking, and between carcinomas and obesity/overweight (*r* = 0.47, *P* = 0.008, and *r* = 0.39 *P* = 0.033, respectively). The prevalence of eyelid skin adenocarcinomas was positively correlated with low fruit intake (*r* = 0.57, *P* = 0.001) and low vegetable intake (*r* = 0.42, *P* = 0.020). A positive correlation was detected between the prevalence of eyelid skin melanomas and obesity/overweight (*r* = 0.35, *P* = 0.055) and low fruit intake (*r* = 0.37, *P* = 0.056). The prevalence of BCC was positively correlated with the provincial prevalence of obesity/overweight (*r* = 0.25, *p* = 0.021). Multivariate analysis showed that provinces with a higher prevalence of obesity/overweight had a higher likelihood of prevalence of eyelid skin total cancers (OR = 1.97, 95% CI = 1.08–3.58), carcinomas (OR = 2.10, 95% CI = 1.15–3.83), and BCC (OR = 1.48, 95%CI = 0.99–2.20) (*P* < 0.05) (Fig. 4).

**Table 2:** Spearman correlation between 3-year prevalence of eyelid skin cancers and the risk factors at the provincial level in Iran

Risk factors	Total tumors		Carcinomas		BCC		Adenocarcinoma		Melanoma	
	**r**	**p**	**r**	**p**	**r**	**p**	**r**	**p**	**r**	**p**
UV exposure	-0.12	0.537	0.11	0.530	-0.18	0.413	-0.15	0.428	-0.20	0.280
Smoking	**0.42**	**0.019**	**0.47**	**0.008**	0.21	0.234	0.23	0.212	0.01	0.966
Alcohol use	0.19	0.294	0.24	0.207	0.31	0.100	0.09	0.636	-0.02	0.923
Low physical activity	-0.22	0.236	-0.18	0.338	-0.13	0.269	-0.14	0.460	-0.08	0.690
Obesity / overweight	**0.37**	**0.045**	**0.39**	**0.033**	**0.25**	**0.021**	0.15	0.407	**0.35**	**0.055**
High cholesterol	0.06	0.759	-0.01	0.995	-0.05	0.815	-0.06	0.717	0.11	0.555
Diabetes	0.05	0.796	0.08	0.671	-0.13	0.496	-0.16	0.384	0.04	0.839
Hypertension	-0.18	0.344	-0.22	0.242	-0.23	0.217	-0.02	0.911	-0.14	0.462
Low fruit intake	*0.34*	*0.069*	0.29	0.110	0.23	0.224	**0.57**	**0.001**	**0.37**	**0.046**
Low vegetable intake	0.14	0.465	0.13	0.474	0.08	0.345	**0.42**	**0.020**	0.03	0.879

Italic rows indicate correlations with borderline *P*- values

*BCC* Basal cell carcinoma

## Discussion

The current study is believed to be the first national report on the epidemiological trend analysis of eyelid skin cancers and the related risk factors in EMR. An increasing trend in overall ASIR of eyelid skin cancers was observed across all age groups, genders, and most morphology types over a 12-year period in Iran. The greatest rise was demonstrated in the ASIR of melanomas. The prevalence of different eyelid skin cancers correlated positively with provincial HDI scores, obesity/overweight, smoking, and fruit and vegetable consumption.

The incidence of eyelid skin cancers was reported in Asia and Europe. Eyelid skin cancers incidence rates were 0.32 in Taiwan (1979–1999), 0.5 in Singapore (1996–2008), and 0.46 per 100000 in South Korea (1999–2016) [7, 12, 25]. Alfaar et al. reported the ASIR of BCC, SCC, and melanoma (6.5, 0.74, and 0.083, respectively) per 100000 German population in 2022 [3]. The ASIR of eyelid skin cancers in the current study (16.22 per 100,000 population) was higher than those reported in Asia and Europe, which might be explained by rapid senescing Iranian population, climate changes [26], duration of our study, and the reference population for standardizing the incidence rate.

An overall increasing trend in the ASIR of eyelid skin cancers, was observed in the current study that was consistent with previous reports [3, 7, 8, 12, 27–31]. This increasing trend may be explained by the global population growth and aging; there has been a 33% global increase in non-melanoma skin cancer cases from 5.8 million in 2007 to 7.7 million in 2017 [32]. On the other hand, Iran was reported as the second fastest aging (≥ 60 years) country in the world between 2015 and 2050 [16]; this pattern might increase the risk of exposure to the eyelid skin cancers risk factors. Gholamnia et al., showed an increase of 0.22% in the population-weighted average values of solar UV radiation during 2005–2019 in Iran [33]. In contrast to other studies, Singapore was the only country in which the ASIR for eyelid skin cancer decreased between 1996 and 2008 [25]. The high level of UV protection, as well as recurrent episodes of atmospheric haze due to forest fires in the mid-1990s, may have contributed to this decline [34].

The majority of eyelid skin cancer morphologies in the current study were carcinomas (88.4%), followed by malignant melanomas (2.9%), and adenocarcinomas (0.7%). BCC was the most common morphology in our study and was similar to the reports from the USA [11]. We observed an increasing trend of eyelid skin cancers ASIR in all morphology types excepting adenocarcinomas; malignant melanoma showed the highest rise during the study period. The increasing trend of BCC was accompanied by a relatively constant trend in malignant melanoma in South Korea (1999–2016) [12]. However, there was no clear geographical or temporal trend associated with malignant eyelid skin cancers in a German study (2009–2015) [3].

In this study, differences in eyelid skin cancers ASIR between provinces may be attributed to multiple factors such as intrinsic (genetics, skin types), behavioral (smoking, diet), environmental (solar radiation, air pollution), urbanization, and socioeconomics. It has been reported that the spatial distributions of solar UV radiation were mainly affected by the latitude in Iran. In addition, at the same latitude, UV radiation values were higher in areas with higher altitudes [33, 35].

According to the United Nations Development Program, Iranian HDI values have increased from 0.565 in 1990 to 0.783 in 2019 [36]. ASIR of total cancers in Iranian women was higher in provinces with higher HDI levels [37]. In the current study, a positive correlation between the ASIRs of eyelid skin cancers and HDI levels was also observed. The GLOBOCAN cancer report in 2018 revealed that the incidence of melanoma and non-melanoma skin cancers (NMSC) was positively correlated with HDI [38]. Public awareness and the practice of skin screenings among different HDI levels may explain the disparity of the eyelid skin cancer ASIR between provinces [39].

The prevalence of total eyelid skin cancers was positively correlated with smoking, obesity/overweight and diet in our study. In a meta-analysis, current and heavy smokers were positively associated with the risk of SCC, and negatively with the risk of BCC and melanoma [40]. It has also been shown that low levels of vitamin A in smokers reduce the level of free radical protection that leads to an increased risk of cutaneous malignancies [41]. Besides, recent studies indicate that nicotine transdermal delivery suppresses the inflammatory response to UVB radiation [42]. Despite the reported inconsistent correlation between smoking and the prevalence of skin cancers, controlling for smoking behaviors are logical to confront the burden of eyelid skin cancers [40, 43].

In the current study, the prevalence of obesity/overweight was positively correlated with total eyelid skin cancers and their morphology subtypes (carcinomas and melanomas). In several studies, excess adiposity was reported as a risk factor for malignant melanoma and NMSC [44–49]. The association between obesity and tumorigenesis has been attributed to chronic low-grade inflammation, elevated levels of growth factors, insulin, and insulin-like growth factor-1 [50, 51]. Nonendocrine tissues respond to insulin and insulin-like growth factor by uptaking glucose and stimulating anabolic pathways that trigger cell proliferation [52]. Obesity is also associated with higher levels of sex hormones and lower levels of their binding proteins, increasing unbound estradiol and cancer risk [53]. The adipocytes in obese individuals produce less adiponectin, which is anti-neoplastic, and more leptin and cytokines, including interleukin 6, interleukin 8, and transforming growth factor-β, which can increase melanoma and NMSC growth and metastasis [54–59]. This association between obesity and melanoma might be influenced by other factors, such as genetics, increased body surface area, gut microbiota dysbiosis, and vitamin D deficiencies [55, 60–62]. Several genetic loci are associated with obesity and melanoma risk, including Agouti signaling protein, interferon regulatory factor 4, peroxisome proliferator-activated receptor-coactivator 1 and vitamin D receptor [63–66]. In addition, obesity has been linked with altered microbiomes that regulate cancer pathogenesis and modulate immunotherapy efficacy in melanoma. The relationship between obesity and BCC is controversial. Karimi et al. and Ferhatosmanoğlu et al. have shown a positive association between obesity and BCC [46, 63]; however, an inverse relationship was observed in other studies [67, 68].

In the present study, the prevalence of eyelid adenocarcinoma was correlated with low fruit and vegetable consumption, and melanoma with low fruit consumption. The antioxidants present in fruit and vegetables inhibit the free radicals generated by UVA and UVB radiation [69]. Vitamins C and E as antioxidants were shown to protect against UV radiation and cancer development by counteracting the increase in reactive oxygen species produced by acute UVB irradiation. Together, these vitamins prevented UVB-induced apoptosis [70]. An Italian case–control study suggested that high citrus consumption was protective against melanoma [71]. In an observational prospective study, the combination of vegetables and fruits was found to decrease the risk of NMSC as opposed to a diet high in meat and fats [72]. However, a recent meta-analysis reported an increased risk of melanoma with citrus fruits and products consumption, which was explained by photoactive mutants in citrus [73].

The current study has several strengths. Globally, there are very few epidemiological studies on the trends of eyelid skin cancers incidence. In addition, based on our knowledge there is no study conducted at the national level in the EMR on these cancers. The present study is one of the very few population-based epidemiological studies that has evaluated the incidence trend of eyelid skin cancers and their relationship to lifestyle risk factors through an integrated INPCR. While the INPCR aims to develop a comprehensive national guideline for population-based cancer registries and produce and publish national cancer statistics, it does not include detailed individual-level data on socioeconomic status and specific risk factors such as family history of cancer. Furthermore, ecology studies cannot be utilized to confirm cause and effect relationships. Therefore, conducting further observational studies that incorporate individual-level socioeconomic and risk factors data is suggested.

## Conclusion

Considering the upward trend of eyelid skin cancers ASIR over a 12-year period in Iran and its burden on the health system, the planning for the improvement of the promotional program is recommended to control obesity/overweight as the main risk factor. An increasing trend of eyelid skin cancer prevalence was consistent with an increase of HDI levels in provinces of Iran. Therefore, an appropriate allocation of screening facilities based on the secular trend and the HDI level might have a positive impact on changing the rising pattern. As the final point, governmental accreditation on accuracy and registering the individual risk factors of the national cancer registry will be helpful.

## Data Availability

Data may be shared to qualified researchers upon reasonable request to the corresponding author.

## Abbreviations

ASIR: Age-standardized incidence rate
HDI: Human Development Index
EMR: Eastern Mediterranean Region
BCC: Basal cell carcinomas
SCC: Squamous cell carcinomas
UV: Ultraviolet
INPCR: Iranian National Population-based Cancer Registry
BMI: Body mass index
EAPC: Estimated annual percentage change
CI: Confidence interval
OR: Odds ratios
ROC: Receiver operating characteristic
AUC: Area under the curves

## References

[1] Siegel RL, Miller KD, Fuchs HE, Jemal A (2022). Cancer statistics, 2022. CA Cancer J Clin.

[2] Kaliki S, Das AV (2020). Ocular and Periocular Tumors in India: an EyeSmart Electronic Medical Record Analysis of 9633 Cases from a Referral Center. Middle East Afr J Ophthalmol.

[3] Alfaar AS, Suckert CN, Rehak M, Girbardt C. The epidemiology of adults' eyelid malignancies in Germany between 2009 and 2015; an analysis of 42,710 patients' data. Eur J Ophthalmol. 2022:11206721221125018. 10.1177/11206721221125018. 10.1177/11206721221125018 PMC9999282 36330713

[4] Gerring RC, Ott CT, Curry JM, Sargi ZB, Wester ST (2017). Orbital exenteration for advanced periorbital non-melanoma skin cancer: prognostic factors and survival. Eye.

[5] Sobanko JF, Sarwer DB, Zvargulis Z, Miller CJ (2015). Importance of physical appearance in patients with skin cancer. Dermatol Surg.

[6] Myers M, Gurwood AS (2001). Periocular malignancies and primary eye care. Optometry.

[7] Lin HY, Cheng CY, Hsu WM, Kao WH, Chou P (2006). Incidence of eyelid cancers in Taiwan: a 21-year review. Ophthalmology.

[8] Quigley C, Deady S, Hughes E, McElnea E, Zgaga L, Chetty S (2019). National incidence of eyelid cancer in Ireland (2005–2015). Eye (Lond).

[9] Lee SB, Saw SM, Au Eong KG, Chan TK, Lee HP (1999). Incidence of eyelid cancers in Singapore from 1968 to 1995. Br J Ophthalmol.

[10] Cook BE, Bartley GB (1999). Epidemiologic characteristics and clinical course of patients with malignant eyelid tumors in an incidence cohort in Olmsted County. Minnesota Ophthalmol.

[11] Baş Z, Sharpe J, Yaghy A, Zhang Q, Shields CL, Hyman L (2023). Prevalence of and Associated Factors for Eyelid Cancer in the American Academy of Ophthalmology Intelligent Research in Sight Registry. Ophthalmol Sci.

[12] Jung SK, Lim J, Yang SW, Jee D, Won YJ (2020). Nationwide Trends in the Incidence and Survival of Eyelid Skin Cancers in Korea. Ophthalmic Epidemiol.

[13] Atlas TC: Human Develpoment Index Transitions. https://canceratlas.cancer.org/the-burden/hdi-transitions/ Accessed 2023.

[14] International WCRF: Cancer rates by Human Development Index. https://www.wcrf.org/cancer-trends/cancer-rates-human-development-index/ Accessed 2023.

[15] BANK TW: Population, total. https://data.worldbank.org/indicator/SP.POP.TOTL?cid=ECR_GA_worldbank_EN_EXTP_search&gclid=EAIaIQobChMI_-q1_-P5gAMVg5eDBx2K9wLaEAAYASABEgJEhvD_BwE&s_kwcid=AL%2118468%213%21665425039372%21b%21%21g%21%21world+bank+projects&view=map (2022). Accessed.

[16] Mehri N, Messkoub M, Kunkel S (2020). Trends, determinants and the implications of population aging in Iran. Ageing Int.

[17] Danaei G, Farzadfar F, Kelishadi R, Rashidian A, Rouhani OM, Ahmadnia S (2019). Iran in transition. Lancet.

[18] Roshandel G, Ghanbari-Motlagh A, Partovipour E, Salavati F, Hasanpour-Heidari S, Mohammadi G (2019). Cancer incidence in Iran in 2014: results of the Iranian National Population-based Cancer Registry. Cancer Epidemiol.

[19] Fritz A, Percy C, Jack A, Shanmugaratnam K, Sobin LH, Parkin DM (2000). International classification of diseases for oncology.

[20] askari pour lahiji H, OTofat Shamsi R (2020). The calculation and evaluation of the human development index of the provinces of Iran in the years 2005, 2010, and 2015. J Hum Capital Empowerment.

[21] Djalalinia S, Modirian M, Sheidaei A, Yoosefi M, Zokaiee H, Damirchilu B (2017). Protocol design for large-scale cross-sectional studies of surveillance of risk factors of non-communicable diseases in Iran: STEPs 2016. Arch Iran Med.

[22] Iran meteorological organization, Iran data portal: https://irandataportal.syr.edu/iran-meteorological-organization. Accessed 2023.

[23] William A. Ryan SB DDV LPdL P, Friel MEG, Karen Hardee, Marianne Haslegrave, Erin, Hasselberg DH, et al. Vlassoff M: THE STATE OF WORLD POPULATION. 2004. https://www.unfpa.org/sites/default/files/pub-pdf/swp04_eng.pdf. Accessed 2023.

[24] Cao G, Liu J, Liu M (2023). Global, Regional, and National Trends in Incidence and Mortality of Primary Liver Cancer and Its Underlying Etiologies from 1990 to 2019: Results from the Global Burden of Disease Study 2019. J Epidemiol Global Health.

[25] Lim VS, Amrith S (2012). Declining incidence of eyelid cancers in Singapore over 13 years: population-based data from 1996 to 2008. Br J Ophthalmol.

[26] Razi S, Enayatrad M, Mohammadian-Hafshejani A, Salehiniya H, Fathali-Loy-Dizaji M, Soltani S (2015). The epidemiology of skin cancer and its trend in Iran. Int J Prev Med.

[27] Anders MP, Nolte S, Waldmann A, Capellaro M, Volkmer B, Greinert R (2015). The German SCREEN project–design and evaluation of the communication strategy. Eur J Public Health.

[28] Tabarés Seisdedos R (2018). Global, regional, and national incidence, prevalence, and years lived with disability for 354 diseases and injuries for 195 countries and territories, 1990–2017: a systematic analysis for the Global Burden of Disease Study 2017. Lancet.

[29] Zhang W, Zeng W, Jiang A, He Z, Shen X, Dong X (2021). Global, regional and national incidence, mortality and disability-adjusted life-years of skin cancers and trend analysis from 1990 to 2019: an analysis of the Global Burden of Disease Study 2019. Cancer Med.

[30] Staples MP, Elwood M, Burton RC, Williams JL, Marks R, Giles GG (2006). Non-melanoma skin cancer in Australia: the 2002 national survey and trends since 1985. Med J Aust.

[31] Wawrzynski J, Tudge I, Fitzgerald E, Collin R, Desai P, Emeriewen K (2018). Report on the incidence of squamous cell carcinomas affecting the eyelids in England over a 15-year period (2000–2014). Br J Ophthalmol.

[32] Fitzmaurice C, Abate D, Abbasi N, Abbastabar H, Abd-Allah F, Abdel-Rahman O (2019). Global, Regional, and National Cancer Incidence, Mortality, Years of Life Lost, Years Lived With Disability, and Disability-Adjusted Life-Years for 29 Cancer Groups, 1990 to 2017: A Systematic Analysis for the Global Burden of Disease Study. JAMA Oncol.

[33] Gholamnia R, Abtahi M, Dobaradaran S, Koolivand A, Jorfi S, Khaloo SS (2021). Spatiotemporal analysis of solar ultraviolet radiation based on Ozone Monitoring Instrument dataset in Iran, 2005–2019. Environ Pollut.

[34] Ilyas M, Pandy A, Jaafar MS (2001). Changes to the surface level solar ultraviolet-b radiation due to haze perturbation. J Atmos Chem.

[35] Zhou Y, Meng X, Belle JH, Zhang H, Kennedy C, Al-Hamdan MZ (2019). Compilation and spatio-temporal analysis of publicly available total solar and UV irradiance data in the contiguous United States. Environ Pollut.

[36] Baumann F (2021). The Next Frontier—Human Development and the Anthropocene: UNDP Human Development Report 2020. Environment.

[37] Alizadeh-Navaei R, Hedayatizadeh-Omran A, Janbabaei G (2017). Cancer incidence pattern in Iran provinces and association with human development index. World J Cancer Res.

[38] Bray F, Ferlay J, Soerjomataram I, Siegel RL, Torre LA, Jemal A (2018). Global cancer statistics 2018: GLOBOCAN estimates of incidence and mortality worldwide for 36 cancers in 185 countries. CA Cancer J Clin.

[39] Lim LT, Agarwal PK, Young D, Ah-Kee EY, Diaper CJ (2015). The effect of socio-economic status on severity of periocular basal cell carcinoma at presentation. Ophthalmic Plast Reconstr Surg.

[40] Arafa A, Mostafa A, Navarini AA, Dong JY (2020). The association between smoking and risk of skin cancer: a meta-analysis of cohort studies. Cancer Causes Control.

[41] Marjan S, Mahmoud D, Mohammad Hassan J, Niloofar S, Abed G, Hamed M. Association of Cigarette Smoking and Serum Concentrations of Vitamins A and E in Men: A Case-Control Study. J Nutritional Sci Dietetics. 2019;5(1–2). 10.18502/jnsd.v5i1.5230.

[42] Mills CM, Hill SA, Marks R (1997). Transdermal nicotine suppresses cutaneous inflammation. Arch Dermatol.

[43] Pirie K, Beral V, Heath AK, Green J, Reeves GK, Peto R (2018). Heterogeneous relationships of squamous and basal cell carcinomas of the skin with smoking: the UK Million Women Study and meta-analysis of prospective studies. Br J Cancer.

[44] Kirkpatrick CS, White E, Lee JA (1994). Case-control study of malignant melanoma in Washington State. II. Diet, alcohol, and obesity. Am J Epidemiol.

[45] Samanic C, Chow WH, Gridley G, Jarvholm B, Fraumeni JF (2006). Relation of body mass index to cancer risk in 362,552 Swedish men. Cancer Causes Control.

[46] Samanic C, Gridley G, Chow WH, Lubin J, Hoover RN, Fraumeni JF (2004). Obesity and cancer risk among white and black United States veterans. Cancer Causes Control.

[47] Gallus S, Naldi L, Martin L, Martinelli M, La Vecchia C (2006). Anthropometric measures and risk of cutaneous malignant melanoma: a case-control study from Italy. Melanoma Res.

[48] Dennis LK, Lowe JB, Lynch CF, Alavanja MC (2008). Cutaneous melanoma and obesity in the Agricultural Health Study. Ann Epidemiol.

[49] Karimi K, Lindgren TH, Koch CA, Brodell RT (2016). Obesity as a risk factor for malignant melanoma and non-melanoma skin cancer. Rev Endocr Metab Disord.

[50] Hopkins BD, Goncalves MD, Cantley LC (2016). Obesity and cancer mechanisms: cancer metabolism. J Clin Oncol.

[51] Avgerinos KI, Spyrou N, Mantzoros CS, Dalamaga M (2019). Obesity and cancer risk: emerging biological mechanisms and perspectives. Metabolism.

[52] Pollak M (2012). The insulin and insulin-like growth factor receptor family in neoplasia: an update. Nat Rev Cancer.

[53] Péqueux C, Raymond-Letron I, Blacher S, Boudou F, Adlanmerini M, Fouque MJ (2012). Stromal estrogen receptor-α promotes tumor growth by normalizing an increased angiogenesis. Cancer Res.

[54] Smith LK, Arabi S, Lelliott EJ, McArthur GA, Sheppard KE. Obesity and the impact on cutaneous melanoma: friend or foe? Cancers (Basel). 2020;12(6). 10.3390/cancers12061583. 10.3390/cancers12061583 PMC7352630 32549336

[55] Clement E, Lazar I, Muller C, Nieto L (2017). Obesity and melanoma: could fat be fueling malignancy?. Pigment Cell Melanoma Res.

[56] Oba J, Wei W, Gershenwald JE, Johnson MM, Wyatt CM, Ellerhorst JA (2016). Elevated Serum Leptin Levels are Associated With an Increased Risk of Sentinel Lymph Node Metastasis in Cutaneous Melanoma. Medicine (Baltimore).

[57] Farag AG, Elnaidany NF, El-Dien MM (2016). Immunohistochemical Expression of Leptin in Non Melanoma Skin Cancer. J Clin Diagn Res.

[58] Singh AK, Arya RK, Maheshwari S, Singh A, Meena S, Pandey P (2015). Tumor heterogeneity and cancer stem cell paradigm: updates in concept, controversies and clinical relevance. Int J Cancer.

[59] Olszańska J, Pietraszek-Gremplewicz K, Nowak D. Melanoma progression under obesity: focus on adipokines. Cancers (Basel). 2021;13(9). 10.3390/cancers13092281. 10.3390/cancers13092281 PMC8126042 34068679

[60] Sergentanis TN, Antoniadis AG, Gogas HJ, Antonopoulos CN, Adami HO, Ekbom A (2013). Obesity and risk of malignant melanoma: a meta-analysis of cohort and case-control studies. Eur J Cancer.

[61] Antoniadis AG, Petridou ET, Antonopoulos CN, Dessypris N, Panagopoulou P, Chamberland JP (2011). Insulin resistance in relation to melanoma risk. Melanoma Res.

[62] Randerson-Moor JA, Taylor JC, Elliott F, Chang YM, Beswick S, Kukalizch K (2009). Vitamin D receptor gene polymorphisms, serum 25-hydroxyvitamin D levels, and melanoma: UK case-control comparisons and a meta-analysis of published VDR data. Eur J Cancer.

[63] Maccioni L, Rachakonda PS, Scherer D, Bermejo JL, Planelles D, Requena C (2013). Variants at chromosome 20 (ASIP locus) and melanoma risk. Int J Cancer.

[64] Granovetter M (2016). IRF4 SNP is predictive of melanoma subtypes. Lancet Oncol..

[65] Shoag J, Haq R, Zhang M, Liu L, Rowe GC, Jiang A (2013). PGC-1 coactivators regulate MITF and the tanning response. Mol Cell.

[66] Köstner K, Denzer N, Müller CS, Klein R, Tilgen W, Reichrath J (2009). The relevance of vitamin D receptor (VDR) gene polymorphisms for cancer: a review of the literature. Anticancer Res.

[67] Lu L, Wan B, Zeng H, Guo J, Li M, Sun M (2023). Body mass index and the risk of basal cell carcinoma: evidence from Mendelian randomization analysis. PeerJ.

[68] Gerstenblith MR, Rajaraman P, Khaykin E, Doody MM, Alexander BH, Linet MS (2012). Basal cell carcinoma and anthropometric factors in the US radiologic technologists cohort study. Int J Cancer..

[69] Katta R, Brown DN (2015). Diet and skin cancer: the potential role of dietary antioxidants in nonmelanoma skin cancer prevention. J Skin Cancer.

[70] Jin GH, Liu Y, Jin SZ, Liu XD, Liu SZ (2007). UVB induced oxidative stress in human keratinocytes and protective effect of antioxidant agents. Radiat Environ Biophys.

[71] Fortes C, Mastroeni S, Melchi F, Pilla MA, Antonelli G, Camaioni D (2008). A protective effect of the Mediterranean diet for cutaneous melanoma. Int J Epidemiol.

[72] Ibiebele TI, van der Pols JC, Hughes MC, Marks GC, Williams GM, Green AC (2007). Dietary pattern in association with squamous cell carcinoma of the skin: a prospective study. Am J Clin Nutr.

[73] Fang X, Han D, Yang J, Li F, Sui X. Citrus Consumption and Risk of Melanoma: A Dose-Response Meta-Analysis of Prospective Cohort Studies. Frontiers in Nutrition. 2022;9. 10.3389/fnut.2022.904957. 10.3389/fnut.2022.904957 PMC9251443 35795586

